# Atypical Cutaneous Fibrous Histiocytoma: An Unusual and Misleading Variant of Fibrous Histiocytoma

**DOI:** 10.1155/2011/612416

**Published:** 2011-07-14

**Authors:** Soumaya Ben Abdelkrim, Colondane Belajouza, Wafa Jomaa, Nadia Beizig, Zeineb Ben Said, Moncef Mokni, Rafia Nouira, Badreddine Sriha

**Affiliations:** ^1^Department of Pathology, Farhat Hached Hospital, Sousse 4000, Tunisia; ^2^Department of Dermatology, Farhat Hached Hospital, Sousse 4000, Tunisia

## Abstract

Atypical fibrous histiocytoma is a distinctive variant of cutaneous fibrous histiocytoma, which is often mistaken histologically for sarcoma and which have a tendency to recur locally and a capacity to metastasize, although very rarely. We report a new case of atypical cutaneous fibrous histiocytoma in a 31-year-old man who presented with a recurrent polypoid nodule on the abdominal wall. The diagnosis was made on the basis of morphological and immunohistochemical findings. We discuss through this case and a review of the literature pathological and evolutive features and diagnostic difficulties of this entity.

## 1. Introduction

Cutaneous fibrous histiocytoma (dermatofibroma) is a frequent and benign neoplasm, which is easily diagnosed by microscopic examination; however, rare variants may be difficult to identify [1]. The atypical variant is distinctly uncommon as some previous reports have described and may be difficult to distinguish from a malignant tumor. This lesion is not well known and merits wider recognition in order to avoid inappropriate treatment [2]. We report a new case of atypical fibrous histiocytoma, and we discuss pathological and evolutive features and diagnostic difficulties of this entity.

## 2. Case Report

**Figure 1:**
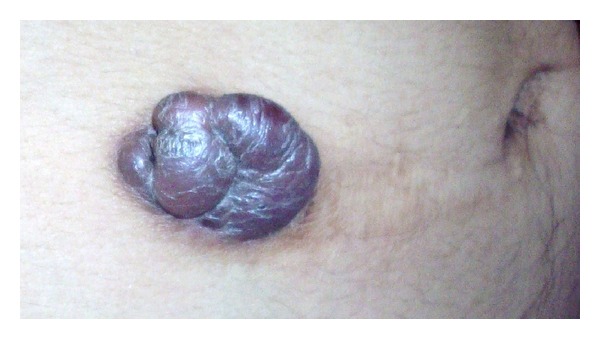
Clinical presentation.

**Figure 2:**
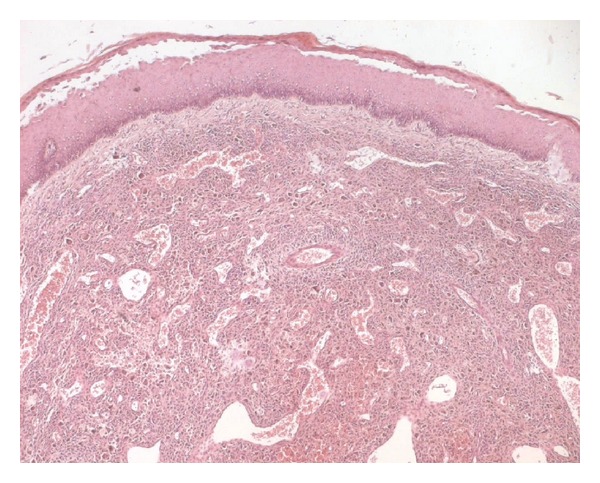
Cellular dermal proliferation of spindle and pleomorphic cells with epidermal hyperplasia, a grenz zone, and prominent blood-filled spaces (hematoxylin and eosin ×40).

**Figure 3:**
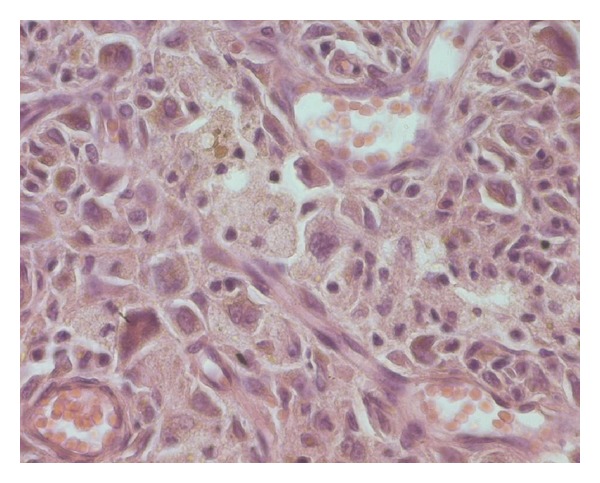
Histiocyte-like and fibroblast-like spindle cells intermingled with atypical mononuclear and giant cells, sometimes with foamy cytoplasm, showing nuclear pleomorphism and bizarre nuclei; a mitosis is seen in the upper right (hematoxylin and eosin ×100).

**Figure 4:**
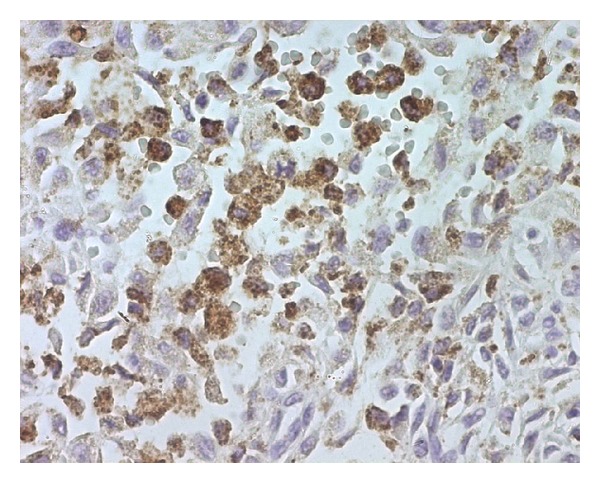
Tumor cells are focally positive for CD68 (immunohistochemistry ×400).

A 31-year-old man presented with an asymptomatic, slowly enlarging, exophytic, brownish nodule of the abdominal wall with surface telangectasia (Figure 1). His past medical and surgical history was significant for a resection of a lesion at the same localization 4 years earlier, which was histopathologically misdiagnosed as dermatofibrosarcoma protuberans. His general condition was good and the rest of systemic examination was normal. The recurrent nodule was totally excised. On macroscopic examination, tumor presented as a well-demarcated nodule of 22 mm in diameter, firm in consistency. On cut surface, it had dark-brown and yellow components. Histological findings demonstrated a well-defined unencapsulated dermal nodule with epidermal hyperplasia, an interposed grenz zone (defined as relatively normal collagen forming a boundary between normal epidermis and a dermal lesion) (Figure 2) and superficial involvement of the subcutis. At the periphery of the lesion, some hyaline large round collagen bundles were seen. The tumor was made of a dense proliferation of predominant histiocyte-like and fibroblast-like spindle cells arranged in interlacing fascicles or a storiform pattern. These cells were intermingled with atypical mononuclear and giant cells, sometimes with foamy cytoplasm, showing large, hyperchromatic, irregular nuclei; we found 4 mitotic figures per ten high-power fields (Figure 3). No atypical mitoses were identified and no necrosis was detected. Prominent blood-filled spaces, numerous siderophages and hemosiderin deposits were noted. Immunohistochemical stains showed focal immunoreactivity for CD68 (Figure 4), while cells were completely negative for S100 protein, HMB45, CD34 and alpha smooth muscle actin. These findings were consistent with the diagnosis of atypical cutaneous fibrous histiocytoma. The revision of the slides of the first tumor revealed the same morphological characteristics and immunohistochemical study was not performed. No recurrence has been detected 3 months after complete removal of the recurrent tumor.

## 3. Discussion

Atypical fibrous histiocytoma is a rare variant of cutaneous fibrous histiocytoma also called pseudosarcomatous fibrous histiocytoma [2] or dermatofibroma with monster cells [3, 4]. It was first described in 1983 [5]. This tumor is seen as a solitary firm cutaneous nodule in a broad age range (5 to 79 years; median: 38 years). Anatomical distribution is wide with most cases occurring in the lower and upper extremities (79%) [6]. Six of 53 lesions (11%) in the series of Kaddu et al. [6] occurred on the trunk, as in our case. Lesion size ranges from 0.4 cm to 8 cm (median 1.5 cm) [6]. Distinctive histological features are pleomorphic, plump, spindle, and/or polyhedral cells with large hyperchromatic irregular nuclei, bizarre multinucleated cells (monster cells), and xanthomatous cells with large prominent nuclei set in a background of classic fibrous histiocytoma, including epidermal hyperplasia, grenz zone, spindle cell areas showing a storiform pattern and entrapped thickened, and hyaline collagen bundles, especially at the periphery [6, 7]. Atypical fibrous histiocytoma may resemble, as in our case, the aneurismal variant of cutaneous histiocytoma but shows in addition numerous pleomorphic cells. The number of mitotic figures ranges from 1 to 15 per 10 high-power fields and atypical mitoses are sometimes noted. There is a spectrum from lesions showing only focal mild pleomorphism to those exhibiting marked pleomorphism. Superficial involvement of the subcutis is seen in one third of the cases. Areas of hemorrhage, siderophages, hemosiderin deposits, and foci of necrosis may be seen [6, 8]. Immunohistologically, the tumor cells are positive for vimentin and negative for S100 protein, epithelial membrane antigen, cytokeratin, and HMB45. Alpha smooth muscle actin, desmin, and CD34 are sometimes focally positive. Positivity for CD68 and factor XIIIa are variable. MiB1 is expressed in less than 10% of the cells [6–11]. Several tumors enter the differential diagnosis including atypical fibroxanthoma, dermal leiomyosarcoma, sarcomatoid carcinoma, nodular melanoma, dermatofibrosarcoma protuberans, angiosarcoma, and pleomorphic fibroma [8]. The criteria for differentiation concern mainly the architectural pattern of the lesion rather than its cytological features [3]. Atypical fibroxanthoma usually presents on sun damaged areas of the head or neck in elderly patients as dome shaped or ulcerated nodule, associated with marked actinic elastosis, and it lacks classic features of fibrous histiocytoma and does not extend into subcutis [6]. The immunohistochemical results in atypical cutaneous fibrous histiocytoma and atypical fibroxanthoma are similar; MiB1 helps to separate these 2 entities from each other as the latter shows a very high proportion of proliferative atypical cells corresponding to the numerous mitoses seen in routine sections [9]. The other differential diagnoses usually lack the classical histologic findings of dermatofibroma and immunohistochemical stains exclude these stimulants: leiomyosarcomais desmin positive, melanoma is positive for S-100 protein, sarcomatoid carcinoma expresses cytokeratin, cutaneous angiosarcoma is positive for CD31 and CD34, and dermatofibrosarcoma protuberans exhibits strong reactivity for CD34 and usually lacks marked pleomorphism [8, 10].

Atypical fibrous histiocytoma has a tendency to recur locally, particularly when incompletely excised. In comparison with classical fibrous histiocytoma, atypical fibrous histiocytoma shows an increased rate of local recurrences (14% versus 1-2%) [6, 12]. No morphologic criteria are useful in prediction of recurrence. Rarely, it may metastasize; in the series of Kaddu et al. [6], 2 patients among 59 developed distant metastases and these 2 cases were not histologically distinct from the group as a whole. Accordingly, this tumor should always be completely excised with clear margins [6].

In conclusion, atypical fibrous histiocytoma is a distinctive but poorly recognized variant of cutaneous fibrous histiocytoma that requires exhaustive histopathological examination and immunohistochemical tests. Recognition of this rare histologic variant is important because it can easily be mistaken for a malignant proliferation, potentially resulting in inappropriate aggressive treatment.
